# Deep Sequencing and Phenotyping in an Australian Tuberous Sclerosis Complex “No Mutations Identified” Cohort

**DOI:** 10.1002/mgg3.70017

**Published:** 2024-10-01

**Authors:** Clara W. T. Chung, Adam M. Bournazos, Lok Chi Denise Chan, Vanessa Sarkozy, John Lawson, Sean E. Kennedy, Sandra T. Cooper, Edwin P. Kirk, David Mowat

**Affiliations:** ^1^ Centre for Clinical Genetics, Sydney Children's Hospital Randwick New South Wales Australia; ^2^ Tuberous Sclerosis Management Clinic, Sydney Children's Hospital Randwick New South Wales Australia; ^3^ Discipline of Paediatrics and Child Health, School of Clinical Medicine, Faculty of Medicine and Health UNSW Sydney New South Wales Australia; ^4^ Kids Neuroscience Centre, Kids Research Children's Hospital at Westmead Westmead New South Wales Australia; ^5^ The Children's Medical Research Institute Westmead New South Wales Australia; ^6^ Specialty of Child and Adolescent Health, Sydney Medical School University of Sydney Camperdown New South Wales Australia; ^7^ NSW Health Pathology Randwick Genomics Laboratory Prince of Wales Hospital Sydney New South Wales Australia

**Keywords:** deep sequencing, genotype/phenotype comparison, mosaicism, tuberous sclerosis complex

## Abstract

Tuberous sclerosis complex (TSC) is a variable multisystem disorder. The “no mutations identified” (NMI) group are reportedly phenotypically milder than those with an identified molecular cause, and often have mosaic or intronic variants not detected by standard sequencing methods.

**Methods:**

We describe the phenotypes in an Australian TSC NMI group (*n* = 18) and a molecular testing strategy implementable in a diagnostic laboratory. Massively parallel sequencing (MPS) of the whole genomic regions of *TSC1* and *TSC2* was performed using DNA extracted from multiple tissue samples per participant.

**Results:**

Our study showed that the phenotype in TSC NMI individuals can be similar to those with heterozygous, particularly *TSC1*, variants. Although neurodevelopmental outcomes can be less severe, the number of organ systems involved was similar to the non‐mosaic groups. A diagnostic yield of 72% (13/18) was achieved, with the majority (10/13) being mosaic variants and the remainder heterozygous variants missed on previous testing.

**Conclusion:**

Testing DNA from multiple tissue samples allowed for validation of otherwise discarded low‐level mosaic variants and detection of mosaic variants by MPS without excessive cost or the need for specialised techniques. Implementing this approach in a diagnostic setting is viable and allows optimal clinical care of patients with NMI TSC.

## Graphical abstract

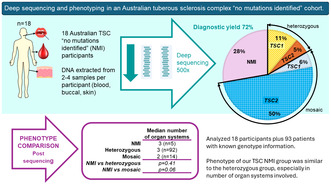

## 1 Introduction

Tuberous sclerosis complex (TSC, [MIM:191100/613254]) is an autosomal dominant condition associated with neurodevelopmental disability and benign tumours in various organs, particularly brain, eye, skin and kidneys. The birth incidence is estimated between 1:6760–1:13520 (Yates et al. 2011; Ebrahimi‐Fakhari et al. 2018).

TSC is caused by heterozygous pathogenic variants in either the *TSC1* [MIM:605284] or *TSC2* [MIM:191092] gene (van Slegtenhorst et al. 1997; European Chromosome 16 Tuberous Sclerosis Consortium 1993). Approximately 10%–15% of individuals who meet diagnostic criteria have no pathogenic variants identified, commonly referred to as the “no mutations identified” (NMI) group (Curatolo et al. 2015; Chung et al. 2017; Tyburczy et al. 2015; Nellist et al. 2015; Martin et al. 2017). Although TSC remains mainly a clinical diagnosis, finding a pathogenic variant may restore reproductive confidence and provides reproductive options for the parents and for the affected individual, as well as potentially allowing access to targeted therapies (Northrup et al. 2021). Timing of testing depends on the reasons for seeking the information. If the individual does not fully meet diagnostic criteria, then a molecular diagnosis might also allow for therapeutic options.

The use of massively parallel sequencing (MPS) at high read depth (deep sequencing) has resulted in an increasing number of the NMI being been found to have mosaic or intronic variants in *TSC1* or *TSC2* (Tyburczy et al. 2015; Nellist et al. 2015; Qin et al. 2010; Ye et al. 2022). However, such sequencing is not widely available in a clinical diagnostic setting. We aimed to describe the clinical features of a cohort of people with a clinical diagnosis of TSC and NMI group and compared their phenotypes with those with an identified variant and previously published NMI groups. Subsequently we sought to develop a high‐yield and cost‐effective testing strategy.

## 2 Methods

### 2.1 Ethical Compliance

Ethics approval was obtained through the Sydney Children's Hospitals Network Human Research Ethics Committee (HREC/18/SCHN/434).

### 2.2 Recruitment

Eighteen patients of the TSC clinic at Sydney Children's Hospital, a tertiary referral clinic, were recruited with informed consent between November 2018 and June 2020. The inclusion criteria were (1) a clinical diagnosis of TSC according to the 2012 diagnostic criteria (Northrup and Krueger 2013) and (2) no disease‐causing variants found by initial diagnostic testing. The only exclusion criterion was the inability to collect samples from at least 2 different sources/tissues. Ninety‐three other patients of the clinic had genetic testing with either a pathogenic or likely pathogenic *TSC1* or *TSC2* variant found. The medical records of these patients were reviewed to form the group for phenotype comparison. Those with variants of uncertain significance were excluded.

### 2.3 Sequencing and Panel Design

A custom Ampliseq for Illumina DNA panel was designed, encompassing the whole genomic region for *TSC1* (NM_000368.5) at chromosome 9:135766735–135820020 and *TSC2* (NM_000548.5) at chromosome 16:2097466–2138716 (GRCh37/hg19). The total combined coverage was 97.96%, with 99.2% coverage over *TSC1* and 96.4% over *TSC2*. All gaps were intronic.

Sequencing was performed on an Illumina MiSeq sequencer at the Ramaciotti Centre for Genomics at the University of New South Wales, Sydney, with a target read depth of 500×, followed by sequencing at 4000× for those that remained NMI.

### 2.4 Variant Analysis

Variant calling was undertaken using BaseSpace DNA Amplicon Application version 2.1.1, Pisces Variant Caller version 5.2.9.23 in somatic mode with a variant frequency threshold of 3%, with alignment using the banded Smith‐Waterman algorithm. Thresholds of 1% and 0.5% were then used for those without a likely causative variant identified. Copy number variant (CNV) calling used the BaseSpace DRAGEN Enrichment Application version 3.8.4 on somatic mode. Variants were visualised on Integrative Genomics Viewer (IGV) version 2.8.0. Variant analysis was done via BaseSpace Variant Interpreter version 2.14 and manual curation of variants. Splicing prediction was performed through Alamut Visual version 2.15.0. Variants were classified according to American College of Medical Genetics and Genomics (ACMG) guidelines (Richards et al. 2015).

### 2.5 RNA Splicing Analysis

Two and a half millilitres whole blood was collected in a PAXgene blood RNA tube (PreAnalytiX) and RNA was isolated using the PAXgene blood RNA kit according to kit instructions. SuperScript IV first‐strand synthesis system (Invitrogen) was used to make cDNA from 500 ng of RNA according to kit instructions. Recombinant Taq DNA polymerase (Invitrogen) and MasterAmp 2X PCR PreMix D (Epicentre Biotechnologies) were added. Thermocycling conditions were 94°C for 3 min, 35 cycles 94°C 30 s, 58°C 30 s, 72°C 90 s/kb, then 72°C 10 min for Recombinant Taq and 94°C for 3 min, 35 cycles 94°C 30 s, 58°C 30 s, 65°C 60 s/kb. Control cDNA was from individuals with genetic variants in an unrelated gene. All PCR products were analysed on a 1.2% agarose gel. Bands were manually excised from an agarose gel with a scalpel and cDNA purified using GeneJET gel extraction kit (Thermo Scientific) according to the Manufacturer's instructions. Purified cDNA and 1 pmol of sequencing primer were subject to Sanger sequencing at the Australian Genomics Research Facility. Sanger sequencing chromatograms were analysed using Sequencher DNA sequence analysis software, Gene Codes Corporation, Ann Arbor, MI USA.

### 2.6 Phenotype Assessment

Phenotype comparisons were made between those with no disease‐causing variants (NMI group), those with mosaic variants (mosaic group) and those with a heterozygous *TSC1* or *TSC2* variant separately and combined as the heterozygous group. Phenotype was ascertained through medical record review. The information obtained included (1) the type of TSC manifestations and (2) the presence and severity of developmental disability (DD), autism spectrum disorder (ASD) and epilepsy. DD was assessed by a developmental paediatrician, assisted by a formal assessment report and the TSC Associated Neuropsychiatric Disorders (TAND) checklist (de Vries et al. 2015). Seizure presence and severity were assessed by a paediatric neurologist. Epilepsy surgery, ketogenic diet, epilepsy‐related mTOR inhibitor use and/or a trial of medicinal cannabis are only considered in individuals with refractory seizures. We used the requirement for one or more of these as objective surrogate markers for seizure severity, categorising these individuals as having “severe seizures.” Fisher's exact test was used for statistical analysis of categorical variables and Kruskal–Wallis test was used for continuous variables.

## 3 Results

### 3.1 The Cohort

The 18 participants were all singleton cases with no known family history. They all met the diagnostic criteria for TSC according to the 2012 and 2021 diagnostic criteria published by the International Tuberous Sclerosis Complex Consensus Group (Northrup et al. 2021; Northrup and Krueger 2013). Their clinical features are summarised in Table S1. Seven (39%) originally had *TSC1* and *TSC2* Sanger sequencing and multiplex ligation dependent probe amplification (MLPA) performed between 2007 and 2009, two of whom had incomplete sequencing coverage of *TSC2*. The remaining 11 (61%) had MPS and MLPA between 2012 and 2019. All prior testing was done on DNA extracted from peripheral blood.

Each participant provided two to four different tissue DNA samples. All provided a sample of peripheral blood, and 17 of the 18 provided a buccal swab. Ten (56%) provided at least 1 skin biopsy sample from TSC‐related lesions (hypomelanotic macule, angiofibroma or Shagreen patch).

Two participants (participants 1 and 14) had a renal AML resected. As these were historical procedures, only formalin‐fixed paraffin‐embedded (FFPE) tissue was available for DNA extraction. Unfortunately, the DNA for both samples did not pass quality control for sequencing and could not be used. For the eight participants with renal AMLs, a urine sample was taken for DNA extraction of urinary sediment. However, the DNA from 6/8 of these samples also did not pass quality control and could not be used.

A total of 47 samples from the 18 participants were successfully sequenced, with consistently high coverage, and amplicon coverage ranging from 424 to 1406 reads (median across all amplicons 853 reads). Seven samples were sequenced in the second run, with mean coverage per amplicon ranging from 3171 to 8908 reads (median across all amplicons 6832 reads).

### 3.2 Diagnostic Yield

**FIGURE 1:**
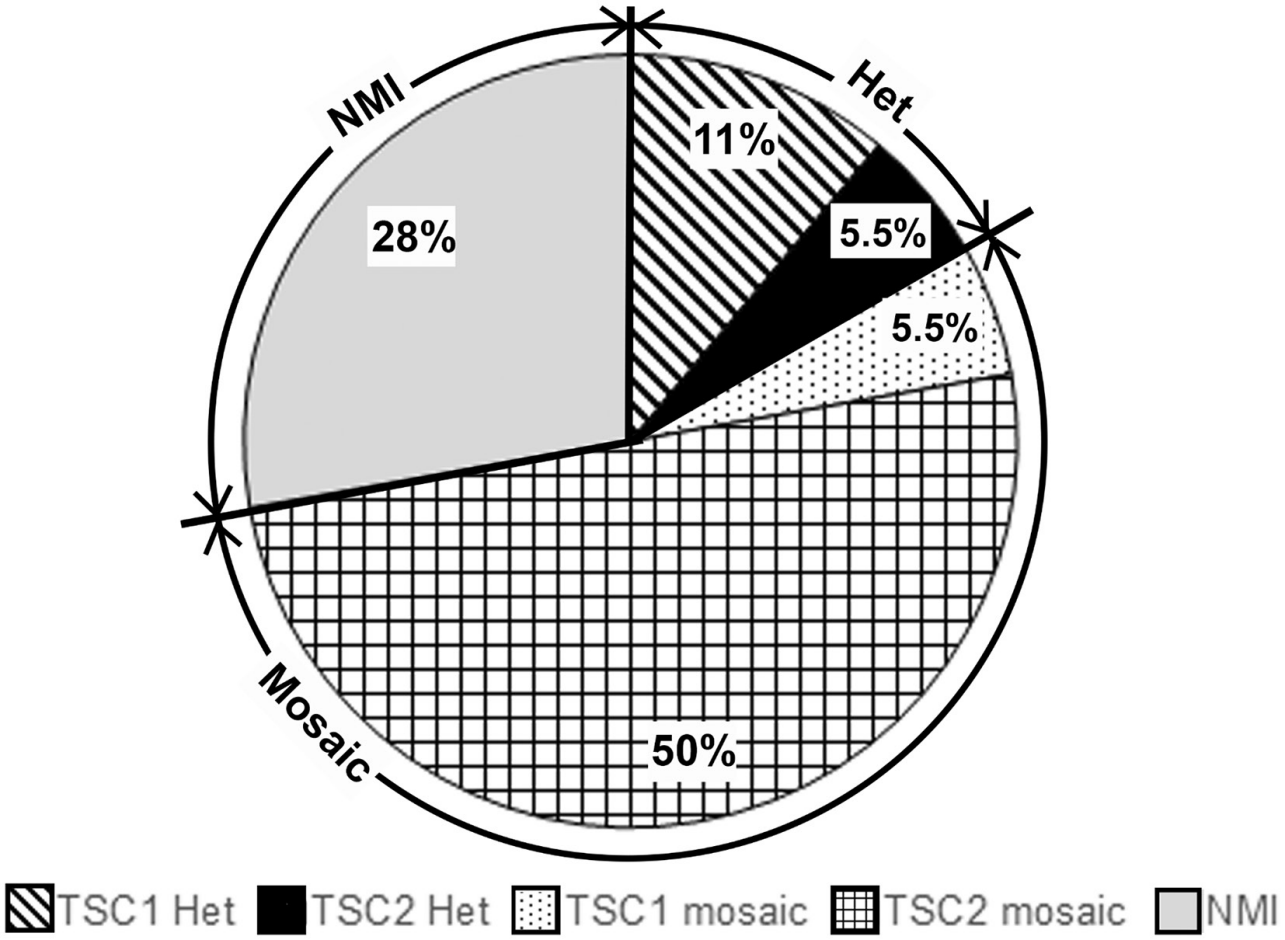
Type of variant found. 13/18 (72%) participants had a disease‐causing variant found on testing. There were 3 previously missed heterozygous variants (16.5%), consisting of 2 *TSC1* and 1 *TSC2* variant. There were 10 mosaic variants (55.5%), consisting of 1 *TSC1* and 9 *TSC2* variants. 5 individuals (28%) had no disease‐causing variants found. Het, heterozygous; NMI, no mutations identified.

Overall, the diagnostic yield was 72% (13/18), of which 3 were heterozygous and 10 were mosaic variants (Figure 1). The variants found are summarised in Table 1. The five persistently NMI participants had deeper sequencing at a depth of 4000×, with no additional diagnostic yield. No deep intronic variants of interest were found. No CNVs were found.

**TABLE 1:** Disease‐causing variants found. Reference sequences: Genome GRCh37/hg19, *TSC1* NM_000368.5, *TSC2* NM_000548.5.

						Blood		Buccal		Skin (HM)		Skin (AF)		Skin (SP)		
	Pt	Gene	Genomic position	cDNA	Amino acid	VAF	Reads	VAF	Reads	VAF	Reads	VAF	Reads	VAF	Reads	Path
**Het**	**2**	*TSC2*	16:g.2136350A > C	c.4819A > C	p.(Thr1607Pro)	50%	676/1409	50%	255/520							LP
	**9**	*TSC1*	9:g.135778042G > A	c.2341C > T	p.(Gln781*)	50%	1619/3256	50%	1563/3108							P
	**13**	*TSC1*	9:g.135796747 T > C	c.737 + 3A > G	p.?	50%	533/1034	50%	743/1584							LP
**Mosaic**	**1**	*TSC2*	16:g.2130180C > T	c.3412C > T	p.(Arg1138*)	4%	16/427	4%	14/380	0.5%	1/230					P
	**3**	*TSC1*	9:g.135781002G > A	c.1963C > T	p.(Gln655*)	5%	34/667	1%	4/621							P
	**5**	*TSC2*	16:g.2138294C > T	c.5227C > T	p.(Arg1743Trp)	4%	35/783	5%	18/419	10%	55/571					P
	**8**	*TSC2*	16:g.2122880C > T	c.2251C > T	p.(Arg751*)	3%	88/2731	2%	48/1981							P
	**11**	*TSC2*	16:g.2135255C > T	c.4594C > T	p.(Gln1532*)	0.2%	1/480	3%	11/407	1%	4/299					LP
	**12**	*TSC2*	16:g.2138294C > T	c.5227C > T	p.(Arg1743Trp)	7%	81/1191	8%	46/580	8%	57/691					P
	**14**	*TSC2*	16:g.2107105G > A	c.775‐1G > A	p.?	3%	14/427	7%	27/366			13%	75/593			P
	**16**	*TSC2*	16:g.2134574dupC	c.4351dupC	p.(Arg1451Profs*73)	18%	88/630					17%	85/500			P
												7%	45/385			
	**17**	*TSC2*	16:g.2112602G > A	c.1361 + 1G > A	p.?	2%	22/1335	3%	28/951							P
	**18**	*TSC2*	16:g.2120571C > T	c.1831C > T	p.(Arg611Trp)	3%	73/2547	2%	12/773					9%	68/800	P

*Note:* Grey shading, sample type not supplied for that participant.

*Indicates a stop codon, as per HGVS nomenclature.

Abbreviations: AF, angiofibroma; Het, heterozygous; HM, hypomelanotic macule; LP, likely pathogenic; Path, variant classification according to American College of Medical Genetics & Genomics criteria; P, pathogenic; Pt, participant; SP, shagreen patch; VAF, variant allele frequency.

The variant allele frequency (VAF) of the mosaic variants detected ranged from 0.2% to 18%. The median VAF in blood was 4% (range 0.2%–18%) and 3% in buccal samples (range 1%–8%). The median VAF in hypomelanotic macules was similar at 5% (range 0.5%–10%). VAF in the other skin samples (angiofibromas and shagreen patches) together was higher (median 11%, range 7%–17%, *p* = 0.03).

### 3.3 Variants

Twelve unique variants were detected, of which one was novel and one had conflicting pathogenicity curation in the literature. The remaining 10 are known disease‐causing variants.

*TSC2*:c.4819A > C, p.(Thr1607Pro) was a novel *de novo* heterozygous missense variant in participant 2, with multiple *in silico* prediction tools predicting a deleterious effect. REVEL score is 0.93 (Ioannidis et al. 2016). This variant is not present in the Genome Aggregation Database (gnomAD) v4.1.0 (Karczewski et al. 2020).

The heterozygous splice site variant found in participant 13—*TSC1*:c.737 + 3A > G—has conflicting variant curations in the medical literature (Jones et al. 1997; Reyna‐Fabián et al. 2020). This variant was not maternally inherited but the father was not available for testing. Reverse transcription polymerase chain reaction (RT‐PCR) was performed on patient mRNA isolated from whole blood. RT‐PCR amplicons were gel purified and Sanger sequenced (Figure S1). The primers used are detailed in Table S2. This detected exon 8 skipping, leading to a frameshift (p.Leu112Asnfs*19), with the resulting mRNA predicted to be targeted by nonsense mediated decay (NMD) or a TSC1 protein lacking 943 of 1164 amino acids from the C‐terminus, including the hamartin domain (Yang et al. 2021). This variant is classified as Likely Pathogenic, following the recommendations of the Australasian Consortium for RNA Diagnostics (Bournazos et al. 2022).

### 3.4 Phenotype of Persistently NMI

The comparison of phenotypic features between the *TSC1*, *TSC2*, mosaic and NMI groups is described in the Table S3. There was no statistically significant difference between the group with mosaic *TSC1* variants compared with mosaic *TSC2* variants, and they were analysed as a group together.

The neurodevelopmental phenotype of the five participants who remained NMI was most like that of the mosaic group. Statistically significant differences were only found in comparing some features in the NMI group and the *TSC2* group, with none found between the NMI and mosaic or *TSC1* groups. These features were the presence of developmental disability (0/5 in the NMI group and 55/76 (72%) in the *TSC2* group, *p* < 0.01) and the presence of seizures (2/5 (40%) in the NMI group and 72/76 (95%) in the *TSC2* group). Both the NMI and mosaic groups had an individual with no abnormal neuroimaging findings (1/5 and 1/14, respectively), whereas all in the heterozygous groups had abnormal neuroimaging findings. Among those with seizures, the age of seizure onset and proportion with severe seizures was not statistically significantly different between groups.

The neurodevelopmental phenotype of the NMI group is most similar to that of the mosaic group. However, when comparing the number of organ systems with clinical features, the NMI group is more similar to that of the heterozygous group. Our NMI group had more organ systems involved that the NMI groups described in previous studies (Table 2) (Tyburczy et al. 2015; Qin et al. 2010).

**TABLE 2:** Median number of organ systems involved compared to other studies. Median (number in group). Statistical calculations based on chi square analysis between the stated groups.

	This study	Ye et al. (2022)	Qin et al. (2010)	Tyburczy et al. (2015)
NMI	3 (*n* = 5)	3 (*n* = 9)	2.5 (*n* = 31)	2 (*n* = 8)
Het	3 (*n* = 92)	2.5 (*n* = 6)	4 (*n* = 5)	3 (*n* = 19)
Mos	2 (*n* = 14)	3 (*n* = 16)	2.5 (*n* = 2)	3 (*n* = 26)
*NMI* versus *het*	*p* = 0.41	*p* = 0.95	*p* = 0.01	*p* = 0.03
*NMI* versus *mos*	*p* = 0.06	*p* = 0.26	n/a^a^	*p* = 0.03

Abbreviations: DD, developmental disability; Het, heterozygous (including both *TSC1* and *TSC2* variants); mos, mosaic; n, number; NMI, no mutations identified.

^a^ Qin *et al*. did not perform statistical analysis for NMI versus mosaic group due to low numbers.

## 4 Discussion

### 4.1 Diagnostic Yield

Our testing achieved a diagnosed yield of 72%, which included 3 previously missed heterozygous variants and 10 mosaic variants below the detection threshold of previous testing. These results have restored reproductive confidence in at least two participants, with one (participant 1) having become pregnant since the study. We were also able to provide genetic counselling and testing in participant 17's daughter who had atypical hypomelanotic macules but did not meet diagnostic criteria for TSC. Deep sequencing and other methods, such as droplet digital polymerase chain reaction (ddPCR), allow for a higher diagnostic yield with detection of variants with lower VAF. In designing and implementing diagnostic assays for mosaic variants, there are trade‐offs that need to be made between cost and yield, with deeper sequencing potentially providing enhanced diagnostic yield but at a higher price. Cost may be a barrier to diagnostic implementation of testing for mosaic variants, potentially acting as a barrier to patients and their families receiving a molecular diagnosis. Previous studies using deep sequencing have used read depths of 1200× to 5000× (Tyburczy et al. 2015; Ye et al. 2022; Giannikou et al. 2019). Our methodology provides a diagnostic yield comparable to these studies in the literature using a target read depth (500×) that is more cost‐effective for a clinical diagnostic laboratory to implement (Tyburczy et al. 2015; Nellist et al. 2015; Qin et al. 2010; Ye et al. 2022; Ogórek et al. 2020). Sequencing and reagent costs in this study were $380 (Australian dollars (AUD)) per sample at 500× compared with $806 AUD per sample at 4000×. This does not account for labour, instrument and laboratory running costs. Sequencing two samples at 500× is more economical than a single sample at 4000×, and the second sample is not required in all cases. In our study, the majority of variants were found in enough reads in blood to be reportable diagnostically. Only 1 case (participant 11) would have required a second sample for confirmation. A stepwise approach of sequencing DNA from blood first, then using a second tissue if required, would further improve cost‐effectiveness.

**FIGURE 2:**
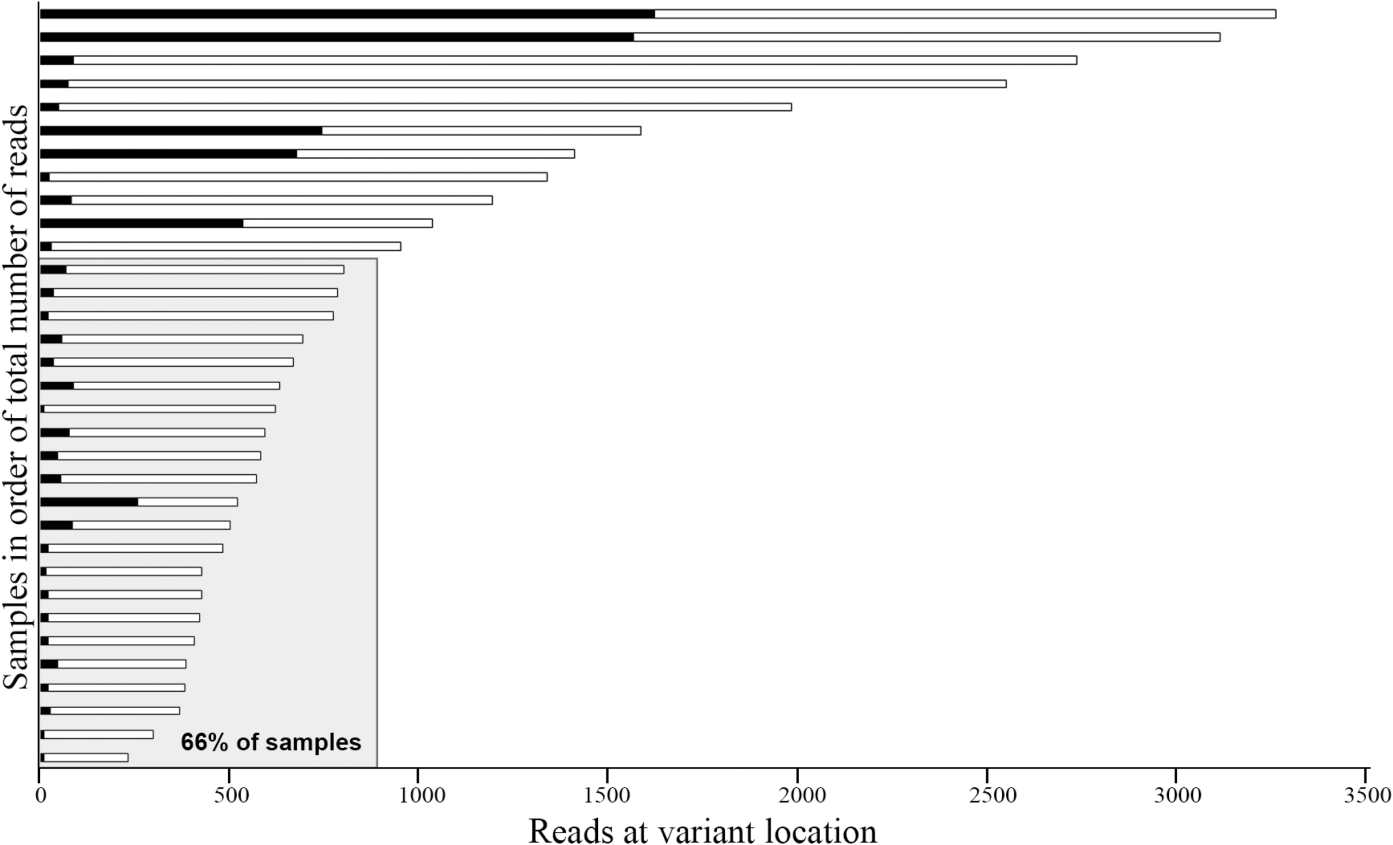
Samples in which a variant was detected arranged in order of total number of reads at location of variant. Number of variant reads in black bars and wildtype reads in white bars. Most (22/33, 66%) variants were detected at 800 reads or fewer (shaded area).

Most variants (66%) were detected at a read depth of 800× or lower, half of which were detected at a read depth of 500× or lower (Figure 2). For samples with read depth over 800×, the variant would likely have been detected even at a read depth of 500–800×, based on VAF. Only one sample had a VAF low enough that a read depth of 500× would lead to fewer than 10 variant reads, suggesting it could have been missed at a lower read depth (2% in participant 18, 22/1335 reads). This suggests that a read depth of 500–800× is sufficient for detecting most mosaic variants, especially as our diagnostic yield was not improved by increasing the target read depth to 4000×. Sequencing multiple samples in the same participant allowed for interpretation of variants present at lower read depths. For example, the variant identified in participant 11 was only seen in 1 of 480 (0.2%) reads in her blood sample. In isolation, this variant would likely have been dismissed as a sequencing artefact. However, the same variant was found at 3% (11/407 reads) in buccal DNA and 1% (4/299 reads) in skin DNA, without being found in other cases, so is likely the disease‐causing variant.

Most variants in *TSC1* and *TSC2* are novel (Peron, Au, and Northrup 2018). However, we only found 1 novel variant in this study. All other variants have previously been published in the medical literature and/or variant databases (Ekong 2021a, 2021b; Landrum et al. 2016).

The effectiveness of mTOR inhibitors as medical therapy for TSC‐related tumours, including renal AMLs, subependymal giant cell astrocytomas (SEGAs) and angiofibromas, has meant that there is a reducing clinical burden from such lesions and less need for surgical resection of tumour tissue (Curatolo and Moavero 2012; Bissler et al. 2013; Franz et al. 2013; Koenig et al. 2018) As a result, most participants (14/19, 74%) did not have any tumour/affected tissue available for testing, which may have reduced our diagnostic yield. Six (32%) participants were on mTOR inhibitors for indications of SEGA (2/6), epilepsy (1/6), renal AMLs (2/6) or lymphangioleiomyomatosis (LAM) (1/6). As medical therapy continues to become the predominant mode of therapy for TSC, there will be fewer individuals with tumour/affected tissue available. So, for a variant detection method to be clinically viable, it needs to have a good diagnostic yield in non‐invasive samples. Where present, variants were found in all blood and buccal DNA samples. If both samples are available, affected tissue is likely not required. But it is still possible that the VAF is too low for detection in blood or buccal DNA. To look for alternate non‐invasive sources of DNA, we trialled the use of urinary DNA. Unlike Ye et al. (2022), we did not find urine to be a reliable source of high quality DNA.

### 4.2 Heterozygous Variants

Of the three participants with previously missed heterozygous variants, we were able to contact two of the original laboratories. In both, the variant was detected in the previous testing, but was not reported. The *TSC2*:c.4819A > C variant was not reported due to the high frequency of benign missense variation in *TSC2*. The *TSC1*:c.737 + 3A > G variant was not reported due to a previous report of it being a “polymorphism” (Jones et al. 1997). The initial testing in participant 9 was done in 2009 on a platform with multiple whole‐exon gaps in coverage. Although the *TSC1*:c.2341C > T had already been reported as a disease‐causing variant in 2005 (Sancak et al. 2005), it may have been missed due to the gaps in sequencing. This highlights the value of reviewing sequencing data or resequencing in those who had testing using older methods.

### 4.3 Mosaic Variants

The majority of the variants found in this study were mosaic, supporting previous studies (Tyburczy et al. 2015; Ye et al. 2022; Peron, Au, and Northrup 2018; Treichel et al. 2020). A threshold of 3% VAF combined with manual visualisation on IGV allowed for detection of all mosaic variants in this study. Using lower VAF thresholds of 1% and 0.5% brought forth numerous sequencing artefacts without increasing the diagnostic yield. In the routine diagnostic setting, a threshold of 3% in at least one tissue (with manual checks of other tissues) appears a reasonable trade‐off between sensitivity of the assay and the workload required for analysis. However, a continued search for variants at lower levels may be warranted in some individuals. Interestingly, almost all the mosaic variants found in this study were in *TSC2*, a higher proportion than that found in some previous studies, but similar to Giannikou and colleague's study (Tyburczy et al. 2015; Nellist et al. 2015; Giannikou et al. 2019). This may have been due to ascertainment bias related to the study being based at a tertiary referral clinic. Those with *TSC1* variants are reported to have less severe clinical manifestations on average and may be less likely to be referred to a tertiary clinic (Curatolo et al. 2015; Farach et al. 2019). This is supported by our previous study that found our clinic has a higher proportion of *TSC2* individuals than reported in the literature (Chung et al. 2017).

The average VAF in blood and buccal DNA were similar, and comparable to that of blood and buccal/saliva DNA found in previous studies (Tyburczy et al. 2015; Giannikou et al. 2019; Treichel et al. 2019). It does not appear that either allows for better mosaicism detection over the other. This may be because leucocytes are the predominant source of DNA in saliva, which in turn is an important contributor to DNA extracted from buccal swabs. DNA extracted from affected skin has consistently been shown to have a higher VAF for detected variants compared to blood and buccal/saliva DNA, and this was found in our group as well (Tyburczy et al. 2015; Giannikou et al. 2019; Treichel et al. 2019; Manzanilla‐Romero et al. 2021). Likewise, the VAF of variants in DNA extracted from angiofibromas and shagreen patches in our study was higher. However, results from DNA extracted from hypomelanotic macules was more in line with that of unaffected tissues such as blood and buccal samples.

### 4.4 Persistently NMI

Five participants remained NMI. Four had variants of interest only present in a single sample at a low VAF, which may represent sequencing artefact. A different panel design or an alternate technique, such as ddPCR, would likely be required to confirm the presence/absence of these variants. There were no deep intronic variants found that were assessed as likely to be disease‐causing. A limitation of this study was that using a small amplicon‐based panel alone did not allow for detection of low‐level mosaic CNVs. However, all samples had previously been assessed with MLPA and no exonic CNVs were identified.

### 4.5 Phenotype

Three previous studies compared the number of organ systems involved in NMI TSC patients compared with heterozygous and mosaic patients (Tyburczy et al. 2015; Qin et al. 2010; Ye et al. 2022). Two found the median number of organ systems involved in the NMI group to be less than the heterozygous group (Table 2). Of note, our study did not show this difference. The groups in the study by Ye and colleagues also did not differ significantly in this regard (Ye et al. 2022). Most likely, this was because these NMI groups had more systems involved than in previous studies.

The clinical features of our NMI group were similar to those reported in the literature, apart from some notable exceptions (Table 3). Interestingly, there was considerable variability in the numbers with DD between the NMI groups of different studies, suggesting that these groups are quite heterogeneous with varying ascertainment. None of our NMI group had DD, which was similar to the findings of Peron and colleagues (5%) and Suspitsin and colleagues (0%), but notably different to the patients studied by Lee and colleagues (78% had DD) (Peron et al. 2018; Suspitsin et al. 2018; Lee et al. 2014). This variability may be related to the age of the subjects, as those with an older group had fewer individuals with DD. The presence of seizures was similar in most studies.

**TABLE 3:** Phenotype of individuals with no mutations identified (NMI) in this study and the published literature.

	This study	Ye et al. (2022)	Peron et al. (2018)	Au et al. (2007)	Sancak et al. (2005)	Dabora et al. (2001)	Camposano et al. (2009)	Lee et al. (2014)	Suspitsin et al. (2018)	*p*
Age, median, y ±SD (range)	20 (14–48)	—	26.5 (2–57)	13.4 ± 13.7	—	9.8 (1–33)	21.8 ± 15.5	—	30	
Age dx, median, y (range)	3 (0–38)	—	18 (0–50)	—	—	—	—	4.3 (0–12)	23.2 (0.75–37)	
FHx (%)	0/5	—	2/21 (10%)	—	—	—	3/21 (14%)	3/9 (33%)	—	0.27
Learning										
DD (%)	0/5	4/9 (44%)	1/20 (5%)	27/70 (39%)	8/22 (36%)	12/22 (55%)	4/23 (17%)	7/9 (78%)	0/8 (0%)	< 0.01
ASD (%)	0/5	1/9 (11%)	1/22 (5%)	—	—	—	—	—	1/8 (13%)	0.75
Neurology										
Seizures (%)	2/5	7/9 (78%)	12/20 (60%)	56/94 (60%)	17/25 (68%)	25/37 (68%)	13/23 (57%)	9/9 (100%)	2/8 (25%)	0.07
Cortical tubers (%)	3/5	8/9 (89%)	21/22 (95%)	49/69 (71%)	14/18 (78%)	19/24 (79%)	18/23 (78%)	8/9 (89%)	0/8 (0%)	< 0.01^a^
SEN (%)	4/5	7/9 (78%)	15/22 (68%)	45/67 (67%)	18/22 (82%)	26/34 (76%)	16/23 (70%)	6/9 (67%)	0/8 (0%)	< 0.01^a^
SEGA (%)	1/5	1/9 (11%)	0/22 (0%)	9/69 (13%)	1/14 (7%)	2/37 (5%)	3/23 (13%)	2/9 (22%)	0/8 (0%)	0.54
Renal										
AMLs (%)	4/5	2/9 (22%)	15/22 (68%)	25/64 (39%)	9/16 (56%)	16/35 (46%)	9/21 (43%)	6/9 (67%)	5/8 (63%)	0.09
Cysts (%)	2/5	1/9 (11%)	6/22 (27%)	20/67 (30%)	5/16 (31%)	6/34 (18%)	4/21 (19%)	—	0/8 (0%)	0.46
Skin, any (%)	5/5	—	—	—	—	—	21/23 (92%)	9/9 (100%)	—	0.53
HM (%)	2/5	3/9 (33%)	15/20 (75%)	75/96 (78%)	17/23 (74%)	26/35 (74%)	—	—	3/8 (38%)	0.01
AF (%)	5/5	4/9 (44%)	17/20 (85%)	50/95 (53%)	14/22 (64%)	21/36 (58%)	—	—	4/8 (50%)	0.04
Shagreen patch (%)	2/5	1/9 (11%)	2/20 (10%)	27/89 (30%)	3/16 (19%)	9/35 (26%)	—	—	0/8 (0%)	0.14
Ungual fibromas (%)	0/5	0/9 (0%)	11/20 (55%)	12/88 (14%)	4/15 (27%)	3/35 (9%)	—	—	1/8 (13%)	< 0.01
CR (%)	2/5	3/9 (33%)	6/22 (27%)	23/68 (34%)	5/15 (33%)	19/34 (56%)	—	3/9 (33%)	1/8 (13%)	0.93
LAM (%)	1/2	0/7 (0%)	5/8 (63%)			0/7 (0%)	4/5 (80%)		3/8 (38%)	0.05
RH (%)	0/5	1/6 (17%)	3/22 (14%)	9/54 (17%)	2/16 (13%)	5/28 (18%)	—	3/6 (50%)	1/8 (13%)	0.46

Abbreviations: —, no data available; AF, facial angiofibromas; Age dx, age at diagnosis; AMLs, renal angiomyolipomas; ASD, autism spectrum disorder; CR, cardiac rhabdomyomas; DD, developmental delay, including intellectual disability; FHx, family history; HM, hypomelanotic macules; LAM, lymphangioleiomyomatosis; RH, retinal harmatomas; SD, standard deviation; SEGA, subependymal giant cell astrocytomas; SEN, subependymal nodules; y, years.

^a^ Not statistically significant without Suspitsin 2018.

Our study was based in a tertiary referral TSC clinic, where many individuals have been known to the clinic since childhood. This may have biased our data as those referred for care at a tertiary centre are likely to be more severely affected, possibly explaining the higher number of features in the NMI group.

## 5 Conclusion

In conclusion, we were able to achieve a good diagnostic yield in our TSC NMI group using amplicon‐based MPS at a target read depth of 500×, suggesting that all TSC NMI individuals should have deep sequencing to look for mosaic variants. We determined that by using multiple samples, a lower target read depth can still result in a good diagnostic yield. This is likely to be more cost‐viable for a diagnostic laboratory to implement. A deep sequencing panel at a read depth of 500× is now clinically available in our local diagnostic laboratory.

## Author Contributions

C.W.T.C. and D.M. conceptualized the project. C.W.T.C., D.M., and E.P.K. designed the study methodology. C.W.T.C. and E.P.K. performed the investigation and data analysis. A.M.B. and S.T.C. performed RNA studies and data analysis. C.W.T.C., L.C.D.C., V.S., J.L., S.E.K. and D.M. collected clinical data for phenotyping. C.W.T.C. wrote the original manuscript draft. All authors reviewed, edited and approved the final manuscript.

## Conflicts of Interest

The authors declare no conflicts of interest.

## Supporting information


Table S1.

Table S2.

Table S3.

Figure S1.

## Data Availability

We confirm that all variants found, including the novel variant, have been submitted to the respective *TSC1* or *TSC2* variant database on Leiden Open Variation Database (https://databases.lovd.nl/shared/genes/TSC1; https://databases.lovd.nl/shared/genes/TSC2). Database variant IDs 0000880205, and 0000880458–000880468.
