# Supplementary material for: Deep Sequencing and Phenotyping in an Australian Tuberous Sclerosis Complex “No Mutations Identified” Cohort
Source: Mol Genet Genomic Med. 2024 Oct 1;12(10):e70017. doi: 10.1002/mgg3.70017 (PMC11443604; doi:10.1002/mgg3.70017)
Supplement: Supplementary file 1 — Table S1. [file MGG3-12-e70017-s002.docx]

| **Pt** | **Sex** | **FHx** | **Age (y)** | **Age at dx** | **Pres. Feat.** | **Sz** | **DD** | **Br** | **Kid** | **Sk** | **He** | **Lu** | **Eye** | **mTOR** |
| --- | --- | --- | --- | --- | --- | --- | --- | --- | --- | --- | --- | --- | --- | --- |
| **1** | F | n | 26 | 9y | HM |  |  | ● | ● | ● |  |  |  |  |
| **2** | F | n | 2 | 0^†^ | CR | ● | ● | ● | ● |  | ● |  |  |  |
| **3** | M | n | 8 | 4y | Sz | ● |  | ● |  | ● |  |  |  |  |
| **4** | F | n | 18 | 2m | AML |  |  |  | ● | ● | ● |  |  |  |
| **5** | M | n | 15 | 2y | Sz | ● | ● | ● |  | ● |  |  | ● |  |
| **6** | F | n | 20 | 3y | Sz | ● |  | ● | ● | ● |  |  |  | ● |
| **7** | M | n | 13 | 1y | Sz | ● |  | ● | ● | ● | ● |  |  | ● |
| **8** | M | n | 1 | 0^†^ | CR |  |  | ● |  |  | ● |  |  |  |
| **9** | M | n | 13 | 0^†^ | CR | ● |  | ● |  | ● | ● |  |  | ● |
| **10** | F | n | 48 | 38y | AML |  |  | ● | ● | ● |  | ● |  |  |
| **11** | F | n | 19 | 12y | HM |  |  |  | ● | ● |  |  |  |  |
| **12** | F | n | 14 | 0^†^ | CR | ● | ● | ● |  |  | ● |  |  |  |
| **13** | M | n | 9 | 10m | Sz | ● | ● | ● |  | ● |  |  |  | ● |
| **14** | F | n | 36 | 32y | AML |  |  | ● | ● | ● |  | ● |  | ● |
| **15** | F | n | 25 | 11y | HM |  |  | ● | ● | ● |  |  |  |  |
| **16** | M | n | 36 | 4y | HM |  |  | ● | ● | ● |  |  |  |  |
| **17** | F | n | 39 | 16y | HM |  |  | ● |  | ● |  | ● |  | ● |
| **18** | M | n | 23 | 1y | Sz | ● |  | ● | ● | ● |  |  | ● |  |

**Supplemental material S1: The clinical features of the study participants.** Pt, participant number; FHx, family history; dx, diagnosis; Pres. Feat., presenting features; Sz, seizures; DD, developmental disability; Br, brain (neuroimaging abnormalities); Kid, kidney; Sk, skin; He, heart; Lu, lung; mTOR, mTOR inhibitor use; F, female; M, male; Y, yes; N, no; y, years; m, months; HM, hypomelanotic macules; CR, cardiac rhabdomyomas; AML, renal angiomyolipoma; ●, present; 0^†^, this individual presented antenatally.
