# Supplementary material for: Deep Sequencing and Phenotyping in an Australian Tuberous Sclerosis Complex “No Mutations Identified” Cohort
Source: Mol Genet Genomic Med. 2024 Oct 1;12(10):e70017. doi: 10.1002/mgg3.70017 (PMC11443604; doi:10.1002/mgg3.70017)
Supplement: Supplementary file 2 — Table S2. [file MGG3-12-e70017-s004.docx]

| Gene | Primer Name | Exon/Intron | Direction | Sequence (5′ - 3′) |
| --- | --- | --- | --- | --- |
| *TSC1* | Ex6F | Exon 6 | Forward | ATTTTTGGCCGTCTGTCATC |
| *TSC1* | Ex7F | Exon 7 | Forward | CGTCTCCTTTTTGCGTTCTC |
| *TSC1* | Ex8F | Exon 8 | Forward | ATCCAAGGACCATGAACTGG |
| *TSC1* | In8R | Intron 8 | Reverse | GATCACATTTTCAATCTCTCGAA |
| *TSC1* | Ex9R | Exon 9 | Reverse | GTGTCAGCATAAGGGCTGGT |
| *TSC1* | Ex10R | Exon 10 | Reverse | TTCAGTTATCAGCCGTGTCG |
| *GAPDH* | Ex3F | Exon 3 | Forward | TCACCAGGGCTGCTTTTAAC |
| *GAPDH* | Ex6R | Exon 6 | Reverse | GGCAGAGATGATGACCCTTT |

**Supplemental material S3: Primer sequences used in RT-PCRs for participant 13.**
