# Supplementary material for: Deep Sequencing and Phenotyping in an Australian Tuberous Sclerosis Complex “No Mutations Identified” Cohort
Source: Mol Genet Genomic Med. 2024 Oct 1;12(10):e70017. doi: 10.1002/mgg3.70017 (PMC11443604; doi:10.1002/mgg3.70017)
Supplement: Supplementary file 3 — Table S3. [file MGG3-12-e70017-s003.docx]

|  |  | NMI | Mosaic | *TSC1* | *TSC2* |
| --- | --- | --- | --- | --- | --- |
| Demographics | | | | | |
| *Male* | | 1/5 (20%) | 6/14 (43%) | 7/16 (44%) | 38/76 (50%) |
| *Current Age*  *(median)* | | 14y-48y  (20y) | 1y-39y  (18y) | 4y-55y  (15y) | 2y-29y ^‡^  (11y) |
| *Age at diagnosis*  *(median)* | | 0^†^-32y  (21m) | 2m-38y  (36m) | 0^†^-27y  (34m) | 0^†^-13y ^‡^  (3m) |
| *Adult (18+)* | | 4/5 (80%) | 7/14 (50%) | 6/16 (38%) | 11/76 (15%) |
| *FHx* | | 0/5 (0%) | 0/14 (0%) | 5/16 (31%) | 3/76 (4%) |
| Developmental disability | | | | | |
| *Any* | | 0/5 (0%) | 2/14 (14%) | 7/16 (44%) | 55/76 (72%)^‡^ |
| *Mild* | | - | 2/2 | 3/7 | 20/55 |
| *Moderate* | | - | 0/2 | 2/7 | 16/55 |
| *Severe* | | - | 0/2 | 2/7 | 19/55 |
| Clinical Features | | | | | |
| *Seizures* | | 2/5 (40%) | 8/14 (57%) | 13/16 (81%) | 72/76 (95%)^‡^ |
| *Autism spectrum disorder* | | 0/5 (0%) | 1/14 (7%) | 3/16 (19%) | 19/76 (25%) |
| *Neuroimaging findings* | | 4/5 (80%) | 13/14 (93%) | 16/16 (100%) | 76/76 (100%) |
| *Renal features* | | 5/5 (100%) | 5/14 (36%) | 4/16 (25%) | 55/76 (72%) |
| *Cardiac features* | | 2/5 (40%) | 3/14 (21%) | 8/16 (50%) | 50/76 (66%) |
| *Skin features* | | 5/5 (100%) | 11/14 (79%) | 13/16 (81%) | 51/76 (67%) |

**Supplemental material S4:** **The phenotype of those in the NMI group compared to those in the mosaic, *TSC1,* and *TSC2* groups.** NMI, no mutations identified; y, years; m, months; FHx, family history of TSC; 0^†^, antenatal presentation; ^‡^ of statistical significance when compared to NMI group. No statistically significant differences between NMI and mosaic groups, or NMI and *TSC1* groups. Comparisons that were of statistical significance only involved comparison of NMI vs *TSC2* group (denoted with ^‡^), and were: Current age (p=.03); Age at diagnosis (p=.05); Number of adults in group (p<.01); Presence of any developmental disability (p<.01); Presence of seizures (p<.01).
