# Supplementary material for: Deep Sequencing and Phenotyping in an Australian Tuberous Sclerosis Complex “No Mutations Identified” Cohort
Source: Mol Genet Genomic Med. 2024 Oct 1;12(10):e70017. doi: 10.1002/mgg3.70017 (PMC11443604; doi:10.1002/mgg3.70017)
Supplement: Supplementary file 4 — Figure S1. [file MGG3-12-e70017-s001.pdf]

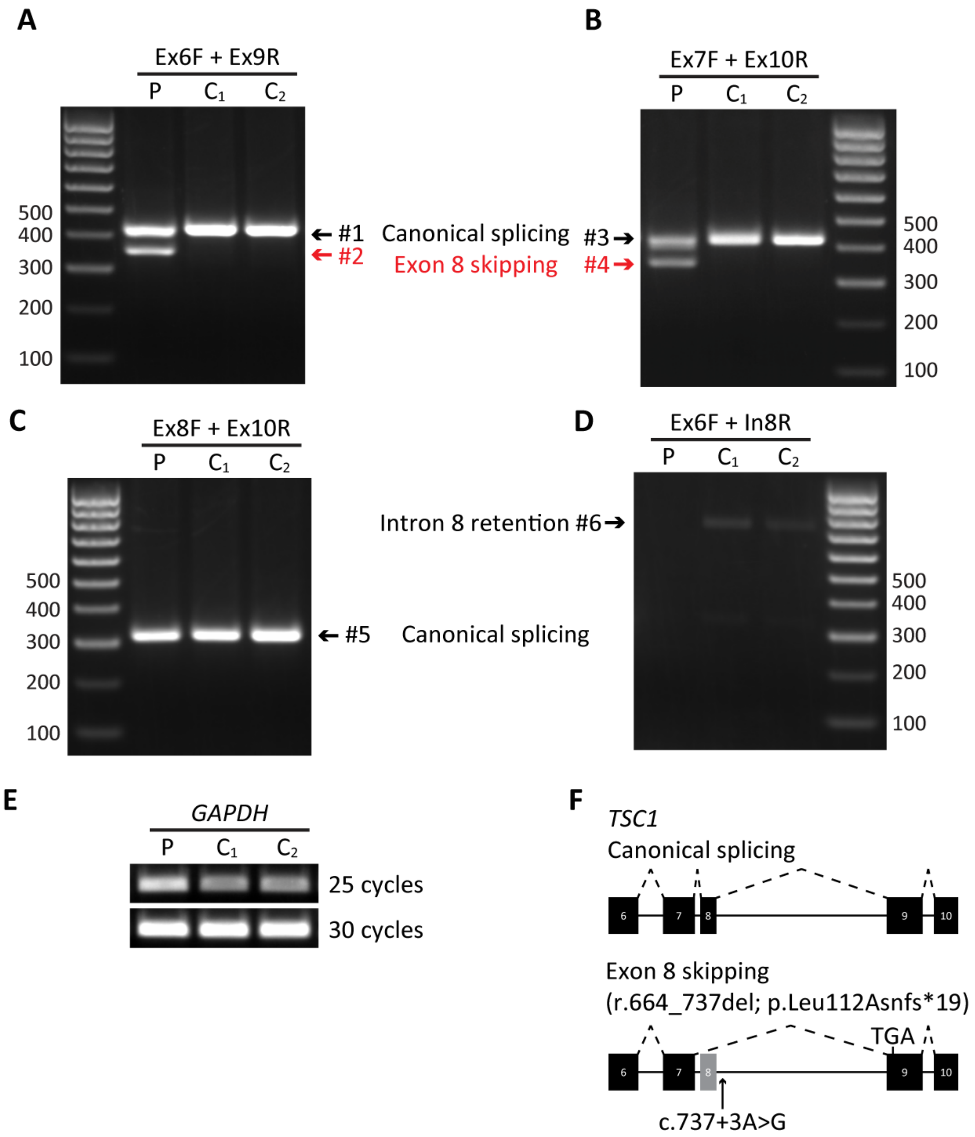

Supplemental material S2: RT-PCR of *TSC1* mRNA isolated from blood for participant 13. Performed by and image supplied by Splicing Diagnostics, Kids Neuroscience Centre, Kids Research. RT-PCR, reverse transcription polymerase chain reaction. Lanes: Proband (P, participant 13), Control 1 (C_1) (male, 10 years old), Control 2 (C_2) (male, 12 years old). **A. & B.** 2 sets of primers flanking the c.737+3A>G variant were used – forward primer in exon 6/reverse primer in exon 9 and forward primer in exon 7/reverse primer in exon 10. Two bands were detected in both sets – bands #1 & #3 show canonical splicing in proband and controls, bands #2 & #4 show exon 8 skipping in proband only. **C.** Using a forward primer in exon 8 and a reverse primer in exon 10, canonical splicing was detected in the proband and controls (band #5). **D.** Using a reverse primer in intron 8, intron 8 retention was not detected (band #6). Use of a downstream cryptic donor site was also not detected. **E.** Amplification of *GAPDH* demonstrates cDNA loading. **F.** Schematic of splicing abnormalities induced by the c.737+3A>G variant.
